# Microsatellite stability and mismatch repair proficiency in nasopharyngeal carcinoma may not predict programmed death-1 blockade resistance

**DOI:** 10.18632/oncotarget.22938

**Published:** 2017-12-05

**Authors:** Xiyi Liao, Liang Zhao, Sangang Wu, Hua Zheng, Haojun Chen, Huan Zhang, ZiJing Wang, Qin Lin

**Affiliations:** ^1^ Department of Radiation Oncology, Xiamen Cancer Hospital, The First Affiliated Hospital of Xiamen University, Teaching Hospital of Fujian Medical University, Xiamen, China; ^2^ Department of Nuclear Medicine & Minnan PET Center, Xiamen Cancer Hospital, The First Affiliated Hospital of Xiamen University, Teaching Hospital of Fujian Medical University, Xiamen, China

**Keywords:** Nasopharyngeal carcinoma, Anti-programmed death-1 antibody, Microsatellite instability-high, Mismatch repair proficiency

## Abstract

The US FDA granted accelerated approval to pembrolizumab for microsatellite instability-high and mismatch repair deficient cancers. The response of programmed death-1 blockade in mismatch repair proficiency (pMMR) colorectal cancer is very poor, however, whether such treatment is effective in pMMR nasopharyngeal carcinoma (NPC) remains unknown.

We report a case of a 51-year-old man with NPC. PET-CT scan revealed a space-occupying lesion in the left lung, and the pathologic result confirmed the occupying lesion originated from NPC. Meanwhile, both immunohistochemistry and PCR revealed that the occupying lesion belonged to pMMR NPC. The lung lesions largely shrunk after chemoradiotherapy. One year later, MRI showed brain occupancy, and brain lesion resection surgery was performed subsequently. The resected tissue was also validated to be the metastatic lesion from NPC. After one month, the patient was examined again by PET-CT, which showed multiple metastases in the liver, pelvis and adrenal gland. Since January 2017, the patient has been treated with pembrolizumab therapy. After five courses of treatment, both PET-CT and blood testing were repeated and demonstrated that metastases and serum Epstein-Barr virus DNA almost completely disappeared.

We provide the first report that pembrolizumab has a confirmed objective response to microsatellite stability and pMMR NPC, and two biomarkers may not be sufficient to identify patients who might be resistant to such treatment in NPC.

## INTRODUCTION

Although various radiotherapy options have a high cure rate in NPC, recurrence and distant metastasis remain key challenges [1]. Therefore, treating this type of NPC is one of the most challenging problems, and a novel effective treatment is imperative. In recent years, several immune checkpoint inhibitors have shown remarkable success in clinical trials, particularly for anti-programmed death-1 (PD-1) antibodies [2–4].As a promising new anticancer strategy, the anti-PD-1 agents have appealed to many clinical trials and shown much remarkable success in the treatment of solid tumours, especially in melanoma, non-small cell lung cancer and renal cell carcinoma [5–7]. However, not all patients with malignant tumours can benefit from this treatment [8, 9]. For instance, only about one-third of patients with melanoma have an objective response to anti-PD-1 antibody therapy, although the response is remarkable [7]. Previous studies have indicated that the clinical response was positively correlated with the expression level of programmed death ligand-1 (PD-L1) [10]. Nevertheless, screening target patients remains controversial because no uniform standard for PD-L1 detection exists [11]. Identifying, before initiation of treatment, which patients are most likely to experience clinical benefit from PD-1 blockade is particularly necessary in the management of tumours considering the expense and low response rates.

Pembrolizumab is the first anti-PD-1 antibody approved by the US FDA [12]. A study of the clinical efficacy and effectiveness of pembrolizumab demonstrated that, in patients with mismatch repair deficient (dMMR) colorectal cancer (CRC), the immune-related objective response rate was 40%, while the corresponding proportion was 0% in patients with pMMR CRC [13]. The response in patients with dMMR non-CRC was similar with that of patients with dMMR CRC[13]. In the 2017 American Society of Clinical Oncology meeting, it was reported that, of 86 patients with advanced dMMR cancers across 12 different tumour types, the objective radiographic response rate of patients to anti-PD-1 antibody was 53% [2]. In May 2017, the FDA granted accelerated approval to pembrolizumab for treating patients with unresectable or metastatic, microsatellite instability-high (MSI-H) or dMMR solid tumours. However, the effectiveness of PD-1 blockade in microsatellite stability (MSS) and pMMR NPC remains undetermined.

We report the case of a 51-year-old man with MSS and pMMR NPC, who showed a “super response” to pembrolizumab treatment.

## CASE PRESENTATION

A 51-year-old Asian male experienced rhinorrhoea with blood in 2012. He was given a clinical diagnosis of nasopharyngeal non-keratotic undifferentiated carcinoma (cT1N2M0, stage III) through nasopharyngoscope and pathological examination. The patient received concurrent chemoradiation and was followed up regularly at our hospital.

In November 2015, a PET-CT scan indicated left lung-occupying lesion, which pathology confirmed originated from NPC. He was given nimotuzumab weekly and docetaxel in combination with nedaplatin 3 weeks for 6 cycles. After the chemotherapy, repeat PET-CT scan revealed the lesion had shrunk, then he received radiotherapy (50Gy/15F) for left lung peripheral metastatic disease. After the left lung peripheral lesions disappeared, he received further radiotherapy (DT 60Gy / 30F) for the left hilar residual because of hoarseness.

**Figure 1:**
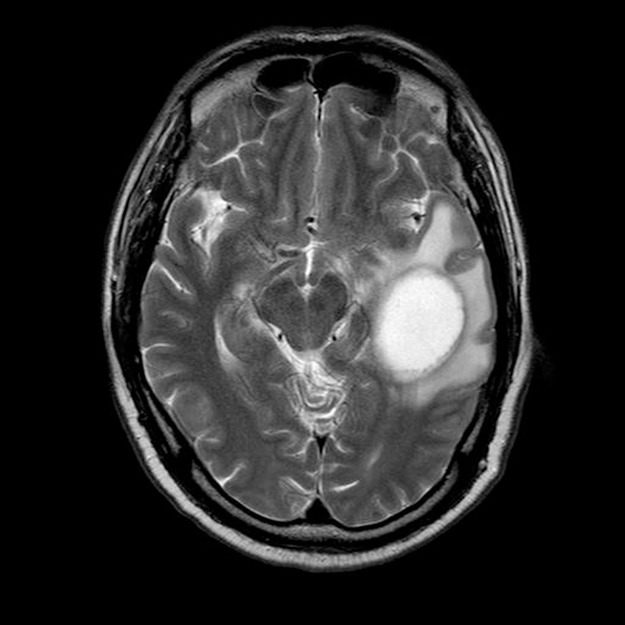
In September 2016, MR scan showed brain occupancy

In October 2016, MR scan showed brain occupancy (Figure 1), and the patient was treated with brain metastases resection surgery. The brain lesion was biopsied and was confirmed to be consistent with NPC. Immunohistochemical analysis showed a small number of CD8-positive lymphocytes in the tumour. The proportion of PD-1-positive cells in CD8-positive lymphocytes was about 70%, while the proportion of PD-L1-positive cells in tumour cells was about 25%. After the surgery, adjuvant radiotherapy (36Gy/12F) was performed.

**Figure 2:**
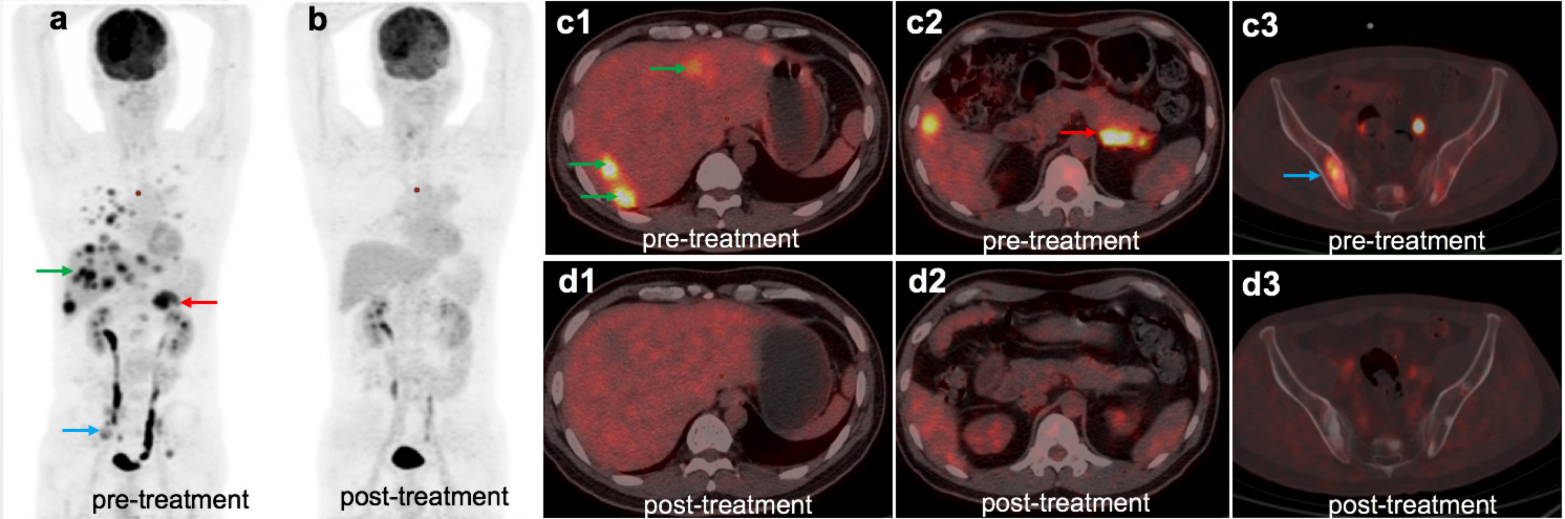
The patient underwent 18F-FDG PET/CT scan before (**a**) and after (**b**) anti-PD-1 treatment, the figure above shows the whole body maximum intensity projection PET and representative PET/CT fused axial images. 18F-FDG PET/CT scan revealed NPC with metastases in multiple organs, including the liver (**c1**), right adrenal (**c2**) and pelvis (**c3**). After receiving 5 cycles of pembrolizumab, all metastatic lesions mostly disappeared (**d1, d2, d3**).

**Figure 3:**
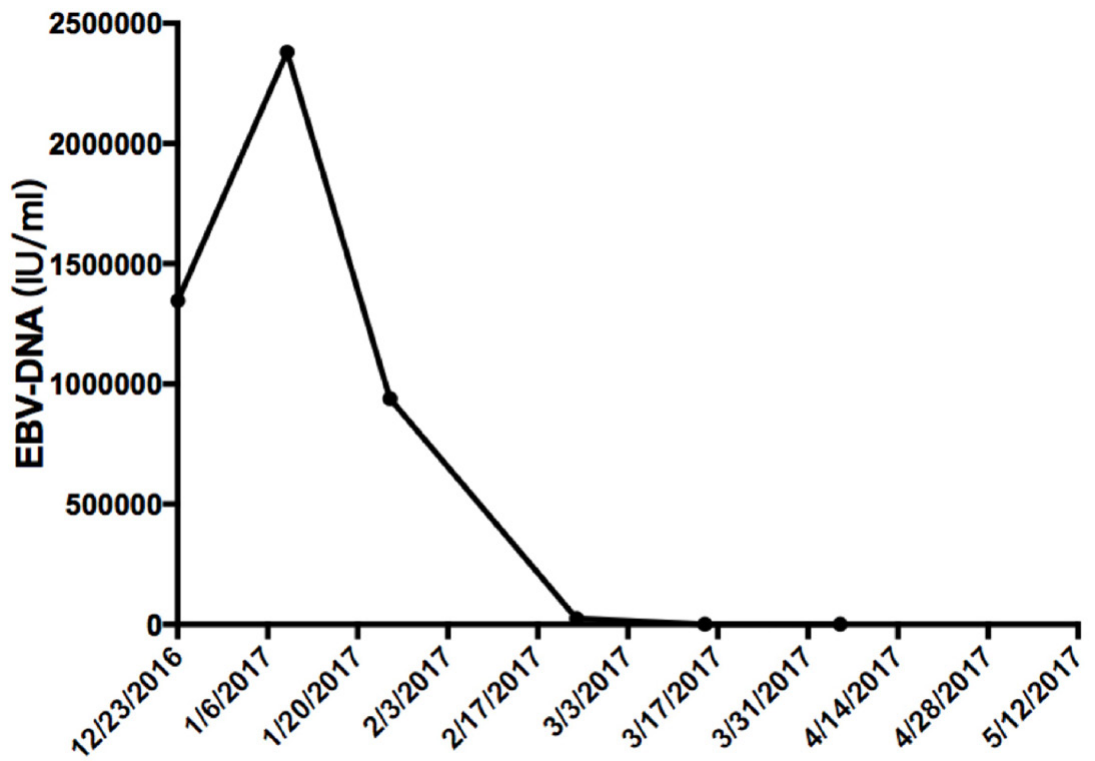
The value of EBV-DNA before and after the treatment of pembrolizumab Since January 10, 2017, the patient has been treated with pembrolizumab therapy.

**Figure 4:**
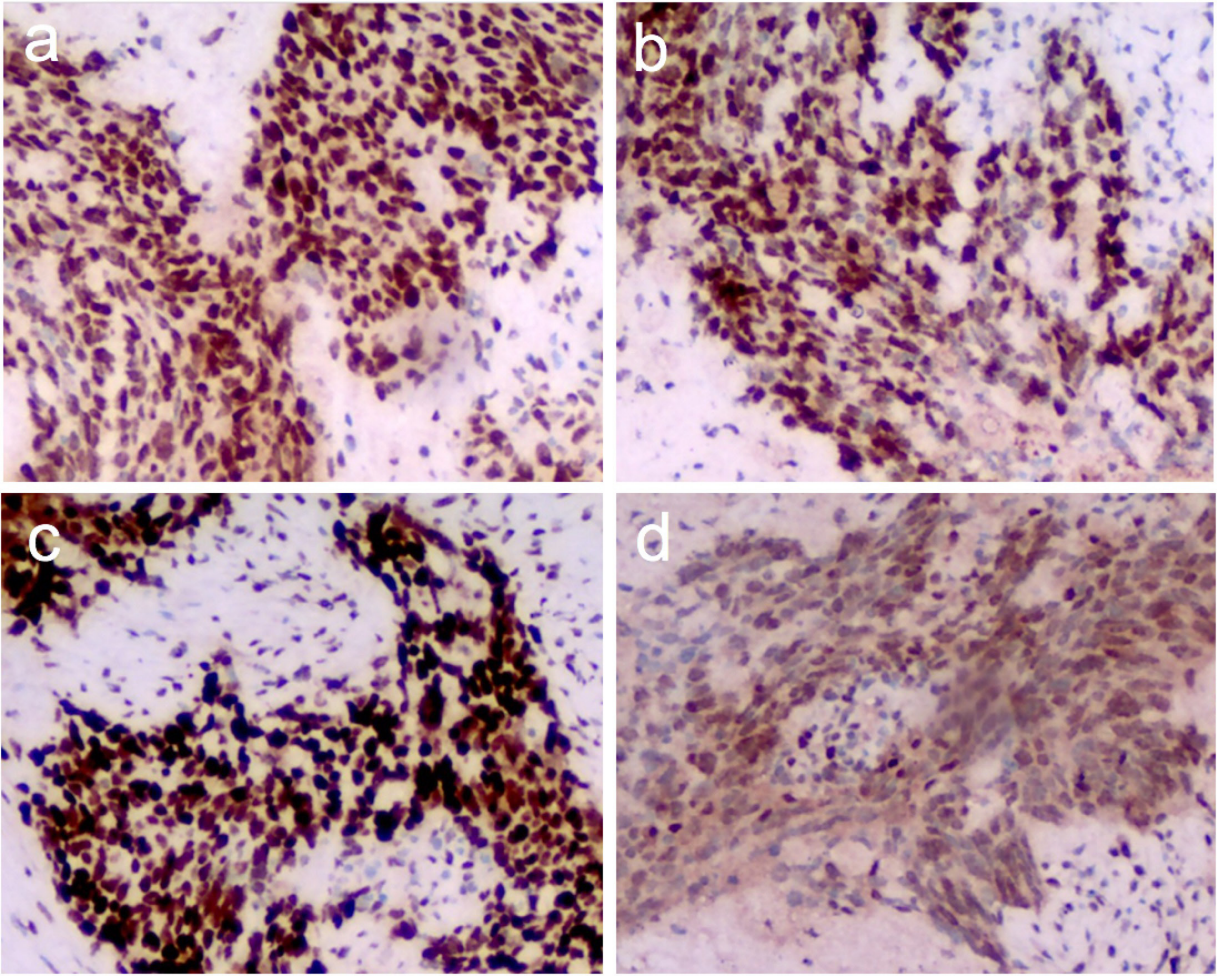
Immunohistochemistry for the mismatch repair (MMR) protein The tumour cells preserved expression of MLH1 (**a**), MSH2 (**b**), MSH6 (**c**), and PMS2 (**d**); original magnification ×100.

**Figure 5:**
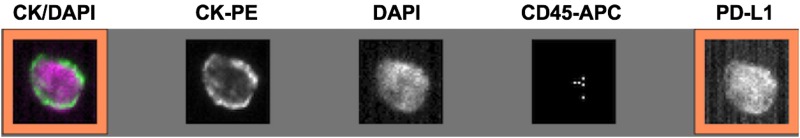
In July 2017, CellSearch-positive CTCs (1 CTC/7.5 ml) in circulation, and this circulating tumour cell is a PD-L1-positive cell CTCs: CK-PE (+), DAPI (+), CD45 (-), Leukocytes: CD45 (+) DAPI (+).

A month later, the patient was examined by PET-CT, and multiple metastases of the liver, pelvis, and adrenal gland were found (Figure 2a, c1-c3). At the same time, the Epstein-Barr virus (EBV)-DNA value in serum was 1.34 × 10 ^ 6IU / ml (Figure 3). Because his pelvic pain was obvious, he was given palliative radiotherapy (DT 20Gy/4F) for pelvic bone metastases. Since January 2017, the patient has received pembrolizumab (100 mg, every 3 weeks) therapy and anti-bone metastasis therapy. After five courses of pembrolizumab treatment, PET-CT and blood testing were repeated and demonstrated that metastases and serum EBV-DNA almost completely disappeared (Figures 2b, d1-d3 and 3). To explore the association of the efficacy of anti-PD-1 antibody with the biomarkers and confirm whether he is an appropriate candidate recommended by FDA (MSI-H or dMMR solid tumour), we used immunohistochemistry (IHC) and polymerase chain reaction (PCR) methods. Both methods demonstrated that the patient had pMMR NPC (Figure 4). Also, the patient presented with CellSearch-positive circulating tumour cells (CTCs) (1 CTC/7.5 ml) in circulation in July 2017, and this circulating tumour cell is PD-L1 positive (Figure 5).

## DISCUSSION

Immunotherapy may be the last hope or the best choice for patients with advanced NPC that had progressed after prior treatment and who have no satisfactory alternative treatment options. PD-L1 is expressed in more than 90% of NPC tumours [14]. It is necessary to study the anti-PD-1 agent and its predictors. More in-depth exploration to help screen the patients most likely to benefit from such therapy is warranted due to expense and other adverse effects.

In May 2017, the FDA approved pembrolizumab for treating patients with MSI-H or dMMR solid tumours. Whether the anti-PD-1 antibody in MSS and pMMR NPC is effective remains unknown. We report the case of a 51-year-old man with MSS and pMMR NPC, who exhibited robust response to pembrolizumab even if he is not an appropriate candidate recommended by the FDA.

So, why does this patient with MSS and pMMR NPC have a “super response” to pembrolizumab? (i) The patient had taken radiotherapy (for primary NPC, lung metastases, and pelvic metastases) before using anti-PD-1 treatment, especially high-dose radiotherapy for pelvic metastases at 5Gy. Ionizing radiation can induce DNA damage and cell death. The dead malignant cells will release tumour-associated antigens and damage-associated molecular patterns (DAMPs) such as high mobility group box 1, nucleotides, or heat shock proteins that can produce an immunogenic response [15, 16]. Thus, radiation therapy can render tumours and their micenvironment more immunogenic [17], which may enhance the efficacy of anti-PD-1 drugs. Preclinical studies have shown that radiotherapy combine with PD-1 blockade can promote anti-tumour immunity [18, 19]. The KEYNOTE-001 trial on previous radiotherapy of pembrolizumab suggested that previous radiotherapy in patients with advanced non-small cell lung cancer results in longer overall survival with pembrolizumab treatment than that seen in patients who did not have previous radiotherapy [20]. (ii) PD-L1 expression is one of the characteristics of EBV-associated tumours, such as NPC [21]. The IHC of our case showed that the proportion of PD-L1-positive cells in tumour cells was about 25%, while the proportion of PD-1-positive cells in CD8-positive lymphocytes was about 70%. Only when PD-1 binds to PD-L1, can PD-1 negatively regulate T cells [22, 23]. The CD8-positive lymphocytes are the major cells that can eliminate the tumour cells, but the PD-L1-positive tumour cells can cause the CD8-positive lymphocytes malfunction. Studies have shown that the clinical response rate of anti-PD-1 drugs corresponds with the level of PD-L1 expression [10, 24, 25].However, different assay methods and cut-off values for PD-L1 positivity would lead to inconsistent classifications of PD-L1 status in some patients [11, 26], resulting in the limitations of the current IHC assessment of PD-L1 expression. IHC is also limited by tumour heterogeneity and sampling variability [27]. Therefore, a substantial unmet need exists for refining the use of PD-L1 expression status as a powerful biomarker for immunotherapy. CTCs can reflect more comprehensive information of individual tumours, which may provide complementary information to identify a candidate for immunotherapy [28]. Our patient's PD-L1 expression was detected both in IHC and CTCs, demonstrating that PD-L1 in tumour cells may be a significant biomarker of immunotherapy response. (iii) MSI positivity are exceedingly rare in NPC patients [29]. If only MSI-H tumour can respond to pembrolizumab, the ratio will be very low in NPC patients. However, in the Keynote 028 trial on the clinical activity of pembrolizumab for treating of NPC, the objective response rate was 25.9% (7 of 27 patients; 95% CI, 11.1 to 46.3) over a median follow-up of 20 months [30]. The above two studies illustrate MSI-H may not be a good predictor in NPC. The biomarkers MSS and pMMR may reflect the resistance of most tumours against PD-1 blockade, but not all solid tumours.

In conclusion, our case report indicates that MSS and pMMR may not reflect resistance to anti-PD-1 therapy in NPC, high-dose radiation therapy before treatment and the expression of PD-L1 in tumour cells may contribute to predicting the response to such treatment. Further research is warranted to explore more precise biomarkers for different tumours.

## Abbreviations

CRC: colorectal cancer
dMMR: mismatch repair deficient
EBV: Epstein-Barr virus
IHC: immunohistochemistry
MSI-H: microsatellite instability-high
MSS: microsatellite stability
NPC: nasopharyngeal carcinoma
pMMR: mismatch repair proficiency
PD-1: programmed death-1
PD-L1: programmed death ligand-1
PCR: polymerase chain reaction
